# A Fluorescent Molecularly Imprinted Polymer-Coated Paper Sensor for On-Site and Rapid Detection of Glyphosate

**DOI:** 10.3390/molecules28052398

**Published:** 2023-03-06

**Authors:** Meng Wang, Jun Qiu, Chennuo Zhu, Yunyan Hua, Jie Yu, Lulu Jia, Jianhong Xu, Jianlin Li, Qianjin Li

**Affiliations:** 1 Jiangsu Key Laboratory for Food Quality and Safety-State Key Laboratory Cultivation Base, Ministry of Science and Technology, Jiangsu Academy of Agricultural Sciences, Nanjing 210014, China; 2 School of Food Science and Pharmaceutical Engineering, Nanjing Normal University, Nanjing 210023, China; 3 State Grid Jiangxi Electric Power Research Institute, Nanchang 330096, China

**Keywords:** molecular imprinting, paper sensor, glyphosate, pesticide residues, rapid detection

## Abstract

Due to the massive use and abuse of pesticides, practices which have led to serious threats to human health, the research community must develop on-site and rapid detection technology of pesticide residues to ensure food safety. Here, a paper-based fluorescent sensor, integrated with molecularly imprinted polymer (MIP) targeting glyphosate, was prepared by a surface-imprinting strategy. The MIP was synthesized by a catalyst-free imprinting polymerization technique and exhibited highly selective recognition capability for glyphosate. The MIP-coated paper sensor not only remained selective, but also displayed a limit of detection of 0.29 µmol and a linear detection range from 0.5 to 10 µmol. Moreover, the detection time only took about 5 min, which is beneficial for rapid detection of glyphosate in food samples. The detection accuracy of such paper sensor was good, with a spiked recovery rate of 92–117% in real samples. The fluorescent MIP-coated paper sensor not only has good specificity, which is helpful to reduce the food matrix interference and shorten the sample pretreatment time, but it also has the merits of high stability, low-cost and ease of operation and carrying, displaying great potential for application in the on-site and rapid detection of glyphosate for food safety.

## 1. Introduction

Glyphosate is a non-selective broad-spectrum organophosphorus herbicide, with high polarity and low volatility [1,2], which is widely used in agriculture, forestry, urban planning, household weeding, and vegetation control [3], particularly in soybeans [4], corn and cotton, due to its low cost and capacity for mass production [5]. The current global production of glyphosate exceeds 800,000 tons per year [6]. Glyphosate is highly resistant to degradation due to the presence of inert C-P bonds in the molecule [7]. Previous studies have suggested that glyphosate poses little harm to animals. However, due to the massive use and abuse of glyphosate, its levels are seriously exceeding the adsorption and degradation capacity of the environment and the substance has been detected in land water and marine water [8,9]. Recent studies have shown that glyphosate has endocrine toxicity, neurotoxicity and cytotoxicity [10], which can affect the human cardiovascular system [6,7]. In March 2015, the International Agency for Research on Cancer (IARC, Lyon, France), a department of the World Health Organization (WHO), classified glyphosate as “probably carcinogenic to humans” [11]. Because of the toxicity of glyphosate, its residual limit standards have been set globally, for example at 0.1–5 mg kg^−1^ for cereals, oils and fruits in China [12]; 0.05–30 mg kg^−1^ for cereals and animal-derived products by the Codex Alimentarius Commission [13]; 20–200 mg kg^−1^ for soybean related products by the US Food and Drug Administration [4]. Therefore, it is urgent for us to develop new techniques to simply and rapidly detect glyphosate residues in food and environment.

The detection of glyphosate, as reported in the literature, mainly relies on traditional chromatographical analytical methods [14]. These have high accuracy and good reproducibility, but their sample pretreatment is complex, professional, and time-consuming [15]; moreover, their detection cost is high, and the detection environment is limited, so it is difficult to achieve rapid detection on site. Rapid detection methods are easy to operate, low-cost, and simple to maintain and run. For glyphosate detection, there are enzyme inhibition methods [16], immunoassay methods [17,18], and electrochemical biosensors [19,20,21]. Although these methods are sensitive, fast and high-throughput, they need biomolecules (e.g., enzymes, antibodies and aptamers) to recognize glyphosate [22], causing issues of poor stability and reproducibility, high-cost and relative long incubation time [11,23].

To solve the above issues of the rapid detection methods, the creation of nanocomposites in polymers to recognize target analytes has attracted many researchers [24,25], and various optical and electrochemical sensors have been developed based on different polymer matrix. A molecularly imprinted polymer (MIP) is a kind of polymer with pre-designed nanocavities for the specific recognition of target molecules. Known as the artificial antibody or receptor, they can be used as substitutes for biological macromolecules [26,27,28,29,30]. For instance, a MIP for glyphosate recognition was created on a gold nanoparticle-modified glass electrode surface, showing a detection limit of 92 ng mL^−1^ and recovery rate of 98%~101% [31]; a paper-based colorimetric analysis device was established using Mn-ZnS quantum dots-embedded MIP targeting glyphosate, giving a detection limit of 2 ng mL^−1^ with a recovery rate of 81–120% [15]. Due to the low cost, ease of carrying, good capillary force, environment-friendly nature and good biocompatibility, paper material has become an excellent carrier for developing rapid detection technology in the fields of food safety and environmental protection [16,32]. Furthermore, coffee ring is a phenomenon when dropping a liquid on the paper. Its formation mechanism is based on the capillary force and liquid evaporation [33], which can be utilized for the enrichment of targets to improve detection sensitivity [34].

In this work, a fluorescent MIP-based paper sensor was designed for on-site and rapid detection of glyphosate. The MIP would be constructed by a catalyst-free imprinting strategy using amino-functionalized silane as the functional monomer, which can interact with glyphosate through its carboxyl and phosphonic acid groups by strong hydrogen bonds. By such strategy, it will be easy to obtain MIPs with high selectivity due to the elimination of the detrimental effect of the catalysts [35,36]. The fluorescent glyphosate-imprinted polymer would be coated on the aldehyde-modified paper surface firmly by Schiff base covalent bonds with the amine-functionalized silane in the pre-polymerization mixture to fabricate a stable and sensitive fluorescent paper sensor. This was expected to display high selectivity, low detection limit and high accuracy.

## 2. Materials and Methods

### 2.1. Reagents and Apparatus

(3-Aminopropyl)triethoxysilane (APTES, >99%), 3-[2-(2-aminoethyl amino) ethylamino]propyltrimethoxysilane (AAPTMS, 95%), tetraethyl orthosilicate (TEOS, >99%), fluorescein isothiocyanate (FITC, ≥96%), and glutaraldehyde were all purchased from Shanghai Macklin Biochemical Co., Ltd. (Shanghai, China). N-[3-(Trimethoxylsilicone) propyl] ethylenediamine (AAAPTMS, 95%) was purchased from Aladdin Industrial Corporation (Shanghai, China). Anhydrous ethanol (≥99.7%) and disodium hydrogen phosphate dodecahydrate (Na_2_HPO_4_·12H_2_O, >99%) were bought from Sinopharm Chemical Reagent Co., Ltd. (Shanghai, China). Glyphosate (96%), 2,4-dichlorophenoxyacetic acid (2,4-D, 96%), chlorpyrifos (96%), and imidacloprid (96%) were provided by the Nanjing Red Sun Group Co., LTD. (Nanjing, China). Purified water was taken from the Hangzhou Wahaha Group Co., LTD. (Hangzhou, China). Double-ring quantitative filter paper was obtained from Hangzhou Wohua Filter Paper Co., LTD. (Hangzhou, China).

Materials morphology was measured on a Zeiss Sigma HD scanning electron microscope (Oberkohen, Germany). UV–vis absorption spectra were recorded with a Yoke UV759 UV/vis spectrophotometer (Shanghai, China). A Darkbox UV analyzer ZF-20D was purchased from Shanghai Guanghao Analytical Instrument Co., LTD. (Shanghai, China). Fluorescent tests for liquid samples were carried on a fluorescence spectrophotometer of FP97pro produced by Shanghai Lingguang Technology Co., LTD. (Shanghai, China). Fluorescence tests for solid samples were performed on an inverted microscope of WSF300 from Guangzhou Microdomain Optical Instrument Co., LTD. (Guangzhou, China).

### 2.2. Synthesis of Fluorescent Monomers

The fluorescent monomers of APTES-FITC, AAPTMS-FITC, and AAAPTMS-FITC were synthesized according to the methods reported in the literature reported [35]. Briefly, FITC (3.9 mg, 0.01 mmol) was dissolved in 2 mL pure ethanol by stirring at room temperature; then, APTES, AAPTMS, or AAAPTMS (0.01 mmol) were added andthe purple color of the solution changed to bright yellow quickly. After stirring for 24 h, the solvent was removed under vacuum evaporation. The products were a thick yellow liquid and used for the polymerization without further purification.

### 2.3. Synthesis of Fluorescent MIPs

The synthetic procedure includes following steps: (1) dissolve the template into the co-solvent of water/ethanol (20 mL/12 mL) under magnetic stirring; (2) introduce the functional monomer into the mixture and stir for 10 min; (3) add the fluorescent functional monomer in ethanol into the mixture and stir for another 10 min at room temperature; (4) drop the crosslinker of TEOS (3 mmol) into the mixture slowly and leave the mixture to undergo stirring at room temperature for 48 h. The detailed contents of the mixture were shown in Table 1. After polymerization, the MIPs were collected by centrifugation (9000 rpm, 10 min) and washed by ethanol to remove the unreacted chemicals. The template in the MIPs was removed by the phosphate buffer (100 mM, pH 8.5). The template removal needed to be washed at least five times, each time using 30 mL phosphate buffer, and so the total volume of the phosphate buffer was about 150 mL. The HPLC method could be used to check if there were glyphosate in the washing solution to confirm whether the template had been removed completely or not. After template removal, the MIPs were washed with pure water three times and ethanol three times in tandem before drying. Non-imprinted polymers (NIPs), the control polymers of MIPs, were prepared under the same conditions as their MIPs, except for the absence of the template.

### 2.4. Preparation of Fluorescent MIP Coated Paper (MIP@P)

The quantitative filter paper was firstly cut into a circle with a diameter of 2.5 cm and modified with aldehyde groups through the APTES. The filter paper was modified with an amino group in 1% APTES/ethanol (6 h), followed by an aldehyde group in 2.5% glutaraldehyde aqueous solution (4 h). Then, the filter paper was immersed into the pre-polymerization mixture before adding TEOS ( reagent, concentration and time used here were the same as that in Section 2.3). After stirring for 15 min, TEOS was added and the polymerization system was stirred for 4 h. Finally, the filter paper was washed with ethanol and water in tandem to remove the unreacted chemicals, and the template in the MIP-coated paper (MIP@P) was removed using the phosphate buffer (100 mM, pH 8.5). The control materials of the NIP-coated paper (NIP@P) were prepared under the same conditions as the MIP@Ps in the absence of the template. The paper sensors were dried and stored in dark conditions at room temperature.

### 2.5. Fluorescence Test of MIP Particles

The MIP particles at certain concentrations (0.01–0.5 mg mL^−1^) were dispersed in a 2 mL phosphate buffer (10 mM, pH 8.0), which was stirred at room temperature. The initial fluorescence signal (F_0_) was measured using a spectrofluorometer (excitation wavelength of 471 nm, emission wavelength of 513 nm) before adding testing molecules. The fluorescence signal (F) was collected after adding the testing molecules into the cuvette. The selectivity of MIP to glyphosate was estimated by the imprinting factor (IF), a value which that was calculated with the following equation:

(1) $$\mathrm{IF}=\frac{{\Delta F}_{\mathrm{MIP}}}{{\Delta F}_{\mathrm{NIP}}}$$

where ΔF_MIP_ and ΔF_NIP_ are the ratios of the fluorescence change on MIP to NIP when meeting glyphosate; the fluorescence change was calculated by the equation as follows:

(2) $$\Delta F=\frac{F_{0}-F_{1}}{F_{0}}$$

where F_0_ is the initial fluorescence intensity and F_1_ is the fluorescence intensity after adding the test molecule.

A series of glyphosate concentrations in the range of 0.5 to 20 µM were added into the testing system containing MIP or NIP. After stirring for 5 min, the fluorescence signal at 517 nm was collected under the excitation wavelength of 471 nm. Each experiment was repeated independently at least three times. Standard curves were created by plotting the fluorescence changes to glyphosate concentrations.

### 2.6. Fluorescence Test of the Fluorescent Papers

The fluorescence signal of the fluorescent paper sensor was measured on a fluorescence-inverted microscope. The fluorescence signal was measured after dropping the sample (20 μL) with different concentrations of glyphosate onto the center point of the paper sensor, which carried different molar amounts of glyphosate (0.1, 0.5, 1, 5, 8, 10 µmol). Each experiment was repeated independently five times.

### 2.7. Specificity Tests of MIP1 and MIP@P

A suspension of MIP1 (0.05 mg mL^−1^) was prepared by dispersing MIP1 particles in a 2 mL phosphate buffer (10 mM, pH 8.0). Then, the testing pesticides of glyphosate, imidacloprid, 2,4-D, or chlorpyrifos were separately added into the system, with a final concentration of 10 μM. The fluorescence signals were measured before and after adding the test molecule to calculate the fluorescence changes. Each experiment was repeated independently at least three times.

The fluorescence signals of MIP@P were measured on the fluorescence inverted microscope and collected before and after dropping different pesticides (glyphosate, imidacloprid, 2,4-D, or chlorpyrifos) onto the center point of the paper sensor. Each experiment was repeated independently five times.

The specificity of MIPs to glyphosate was estimated by the cross-reactivity factor that was calculated by the following equation according to our previously reported method [35,37].

(3) $$\mathrm{Cross-reactivity}\;\mathrm{factor}=\frac{{\Delta F}_{\mathrm{MIP}\left(\mathrm{glyphosate}\right)}}{{\Delta F}_{\mathrm{MIP}\left(\mathrm{other}\;\mathrm{testing}\;\mathrm{molecule}\right)}}$$

where ΔF_MIP(glyphosate)_ and ΔF_MIP(other testing molecule)_ are the ratios of the fluorescence change on MIP, assessed using glyphosate and other testing molecules to the carry out the experimental tests, respectively.

### 2.8. Application in Real Samples

Tap water and soybean samples were selected for testing the detection accuracy of the MIP@P by measuring the recovery rates of the spiked glyphosate. The sample pretreatment procedure was performed according to the method the literature reported [38]. Three parallel trials were used to produce the average value in order to obtain the detection result.

## 3. Results and Discussion

### 3.1. Preparation of MIP@P

**Figure 1:**
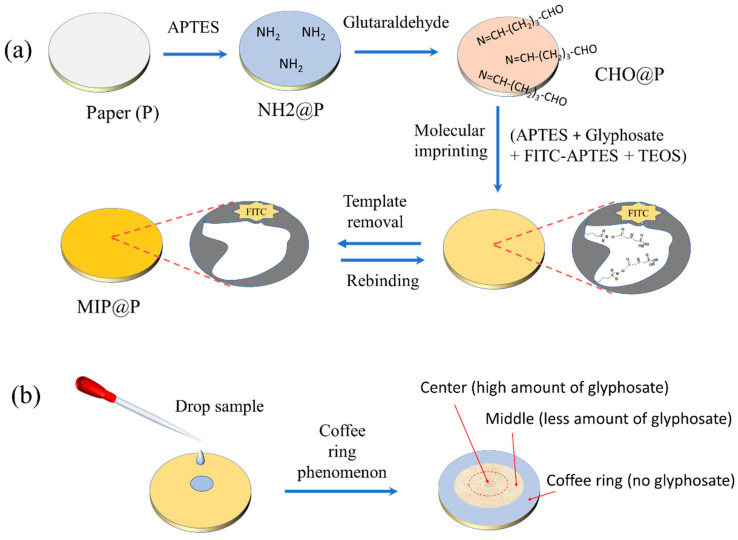
Preparation of fluorescent MIP@P for specific recognition of glyphosate (**a**) sensitively based on the coffee ring phenomenon (**b**).

To prepare a more stable MIP-coated paper sensor, aldehyde groups were modified onto the paper surface to react with the amino group residues in MIP through the Schiff base reaction. As shown in Figure 1a, the amino groups were firstly modified on the paper by APTES to make NH2@P, which was further grafted with aldehyde groups through the covalent reaction between the amino groups and aldehyde groups in glutaraldehyde to obtain CHO@P. Then, a catalyst-free imprinting strategy was used for imprinting glyphosate onto the paper surface [39]. Because the residual aldehyde groups on the paper can react with the abundant amino-functionalized silane in the pre-polymerization mixture through the Schiff base reaction, the final MIP layer could be covalently and stably modified onto the paper. Amino-functionalized silane was used as the functional monomer and also acted as the initiator to catalyze the sol–gel polymerization, and FITC was used as the fluorophore to report the recognition event quantitatively. The molar ratio of template molecule, functional monomer and crosslinker was set as 1:6:30, based on the optimized result reported in the literature [35,36]. Finally, the fluorescent glyphosate-imprinted polymer was coated onto the paper surface by immersing the paper into the polymerization mixture directly. After template removal, the fluorescent paper sensor of MIP@P was obtained. In addition, a very simple analytical method (Figure 1b) was established by the use of the coffee ring phenomenon. By dropping the sample at the center of the paper sensor, the target glyphosate was selectively captured and enriched at the center, and the interferences were brought to the paper edge by the capillary force, which is beneficial for eliminating sample matrix interference.

**Figure 2:**
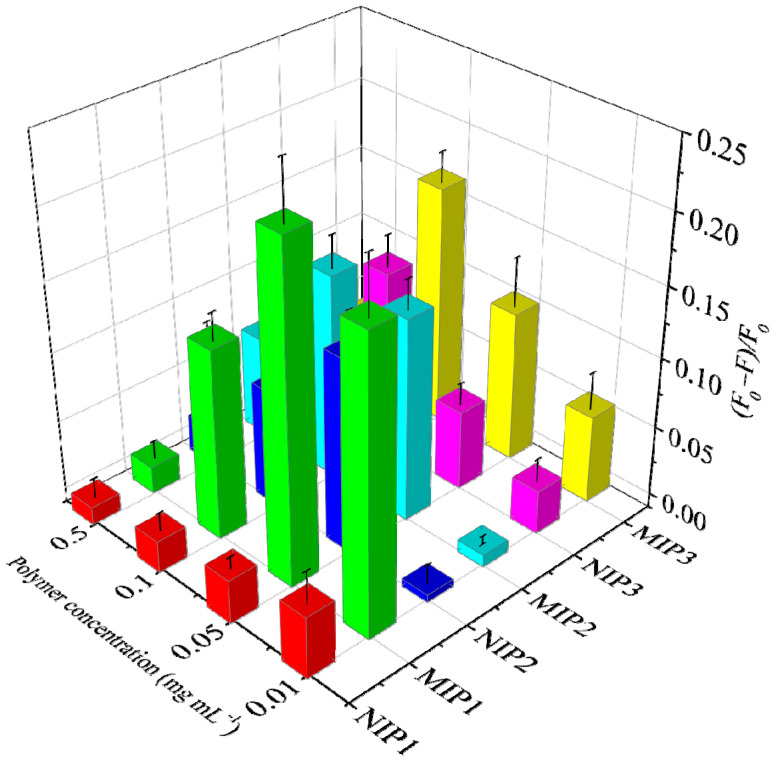
Fluorescence response of the MIPs (MIP1, MIP2 and MIP3) and NIPs (NIP1, NIP2 and NIP3) to glyphosate (10 μM) at different concentrations.

To investigate which amino-functionalized silane is more suitable for imprinting glyphosate, three MIPs, together with their corresponding NIPs, were prepared by using APTES, AAPTMS or AAAPTMS as the functional monomers independently (Table 1). Their selectivity was evaluated by the popular IF values, where a higher IF value means that the MIP has better selectivity [39]. From Figure 2, it can be seen that MIP1 gives a better selectivity (IF = 1.5–7.6) than MIP2 (1.0–3.5) and MIP3 (1.3–4.4) at most polymer concentrations. Such results indicate that different amino-functionalized silanes do can affect the selectivity of MIP [33]. However, the exact reason for this was not clear, but it will be an interesting question to explore by performing more experiments, including molecular simulation. Moreover, it was found that the selectivity evaluation based on IF values can be significantly affected by the polymer concentration because the recognition event reporter is dependent on molecular diffusion in essence [40]. As the IF value of MIP1 at the concentration of 0.05 mg mL^−1^ showed the highest IF value (7.6), this experimental condition was chosen to perform the specificity tests, and the pre-polymerization mixture of MIP1 was employed for preparing the fluorescent MIP-coated paper sensor.

### 3.2. Characterization

**Figure 3:**
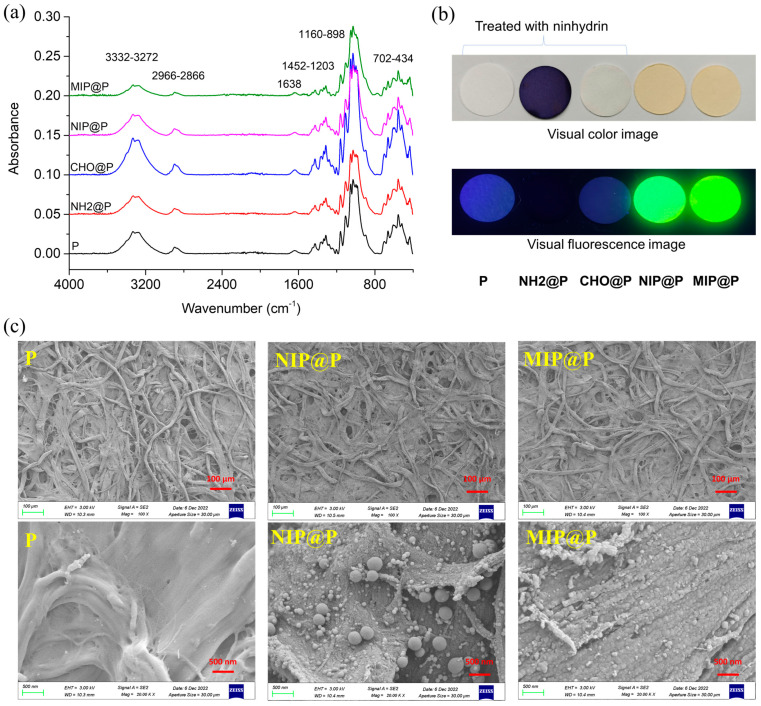
(**a**) IR spectra of P, NH2@P, CHO@P, NIP@P and MIP@P; (**b**) Visual color and fluorescence (excitation light of 365 nm) images of P, NH2@P, CHO@P, NIP@P and MIP@P, the first three paper materials were treated with ninhydrin in ethanol; (**c**) SEM images of P, NIP@P and MIP@P.

All the paper materials were firstly characterized by IR to check if every synthetic step was successful. As shown in Figure 3a, it is surprising to find that all the five papers display almost the same IR peaks, except for their different absorbance intensity, suggesting the IR signals of the supporting paper covered or overlapped with those of the post-modified materials. The characteristic IR peak groups of 3332–3272 cm^−1^, 2966–2866 cm^−1^, 1638 cm^−1^, 1452–1203 cm^−1^, 1160–898 cm^−1^, and 702–434 cm^−1^, were derived from the chemical groups vibration of OH/NH (stretching), CH (stretching), OH/NH (bending), CH (bending), C-O/Si-O (stretching), and OH\NH\CH (bending), respectively, most of which can be attributed to the chemical groups contained in cellulose.

Simple and low-cost methods were used for checking the synthetic performance. One is ninhydrin-based color detection of amine or amino groups. From Figure 3b, it is very clear to see that, when the paper (P) before being grafted with amino groups was treated with ninhydrin, the paper remained its original white color and blue fluorescence; when using ninhydrin to treat NH2@P, the paper showed a dark blue color and no fluorescence, demonstrating that APTES was successfully modified on the paper. Then, the NH2@P was reacted with excessive glutaraldehyde to prepare CHO@P, which was treated with ninhydrin and showed white color, not blue color, and blue fluorescence, confirming that all the amino groups on NH2@P had reacted with the aldehyde groups and that CHO@P had been obtained successfully. In addition, it was also easy to judge the MIP or NIP has been grafted onto CHO@P evenly in view of their even yellow color images or yellow-green fluorescence (Figure 3b).

The morphologies of MIP@P and NIP@P, as well as the naked paper, were characterized by SEM (Figure 3c). It was clear to see both were MIP and NIP coated onto the paper surface successfully and firmly because their surface became rough. After polymer coating, the cellulose skeleton structure of the paper still maintained three-dimensional structure, and the coating layer was very thin, being less than 1 µm. In addition, it could be seen that the coating layer on MIP@P was more uniform than that on NIP@P, which also contains some sub-microspheres and nanospheres.

### 3.3. Selectivity and Specificity of MIP@P

**Figure 4:**
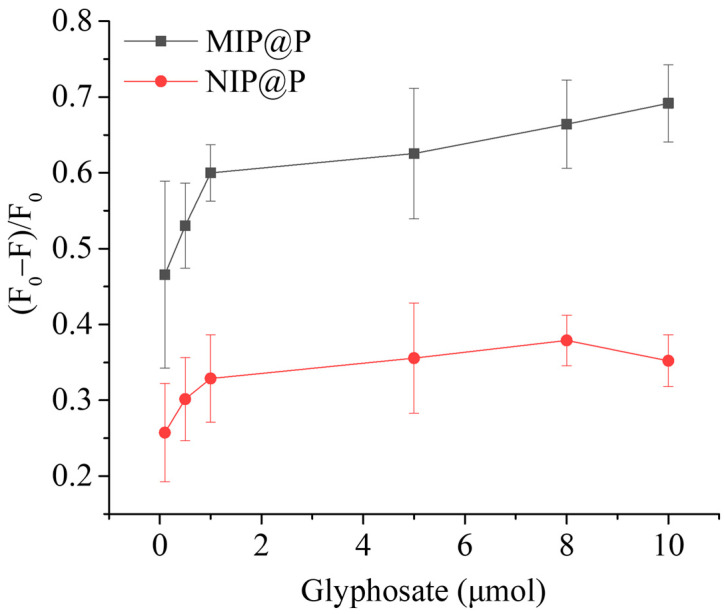
Fluorescence-responsive behaviors of MIP@P and NIP@P when compared to different amounts of glyphosate.

The fluorescence-responsive behavior of MIP@P was investigated, as well as its control material. From Figure 4, it is clear to see that MIP@P exhibited a larger fluorescence change than NIP@P to glyphosate at all testing molar amounts, giving IF values in the range of 1.8–2.0, lower than most of the glyphosate imprinted sensors shown in Table 2, particularly electrochemical sensors (IF = 2.8–14.5), but comparable with the fluorescent sensors (IF = 1.9–2.9) [41,42,43].

**Figure 5:**
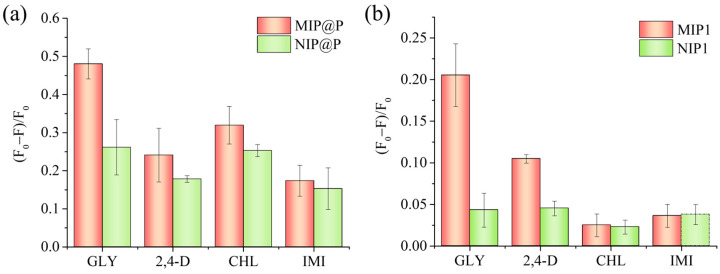
Fluorescence response of MIP@P and NIP@P (**a**), and MIP1 and NIP1 (**b**) to the testing pesticides. GLY, glyphosate; 2,4-D, 2,4-dichloro-phenoxyacetic acid; CHL, chlorpyrifos; IMI, imidacloprid.

To further study the molecular recognition capability of MIP@P, three commonly used pesticides (imidacloprid, 2,4D, and chlorpyrifos) were selected to evaluate the specificity of MIP@P and MIP1, the latter of which was used as a positive control material. As shown in Figure 5a, it could be seen MIP@P showed the most fluorescence-responsive signal to glyphosate among the four testing pesticides, giving the cross-reactivity factors of 2.0 (2,4-D), 1.5 (CHL), and 2.8 (IMI). These values were lower than those of the interaction between MIP1 and glyphosate (Figure 5b), which were calculated to be 2.0 (2,4-D), 8.2 (CHL), and 5.7 (IMI). Such reduced specific recognition capability phenomenon, when changing MIP particles to be paper-supported MIP layers, could be explained by the increased non-specific fluorescence response, which was confirmed by the larger fluorescence response of NIP@P than NIP1 (Figure 5).

### 3.4. Application in Real Samples

To investigate the applicability of the developed fluorescent MIP-coated paper sensor in real samples, we prepared tap water and soybean samples which were spiked with glyphosate with different concentrations. After adding the samples onto the fluorescent paper sensor, the result could be obtained within 5 min.

**Figure 6:**
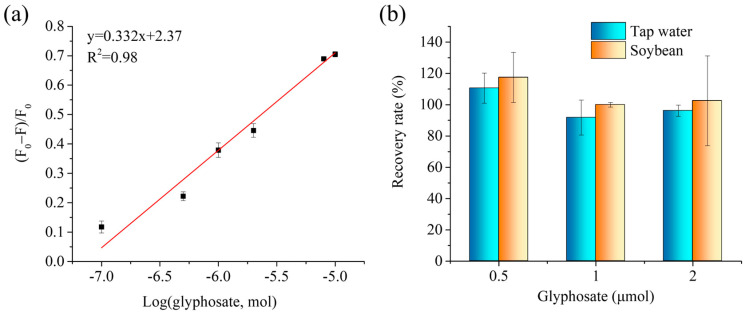
(**a**) Linear curve created by plotting fluorescence response of MIP@P towards glyphosate to its molar amount. (**b**) Spiked recovery rates of the developed fluorescent MIP@P sensor for the detection of the spiked glyphosate in tap water and soybean samples.

Because the liquid sample dropped onto the paper center will spread to the paper side, during which time the liquid will evaporate quickly, it is hard to maintain the analyte concentration at a certain value. However, the molecular molar amount is fixed. Moreover, after the analytes were captured by the MIP@P, the liquid samples could be dropped repeatedly onto the paper to enlarge the fluorescence signal change. To accurately describe the performance of this fluorescent MIP paper sensor, in this manuscript we therefore used the analyte amount instead of analyte concentration to show the analytical parameters, including the limit of detection and linear detection range. From Figure 6a, it can be seen that the paper sensor displays a linear curve in the range of 0.5–10 μmol with a detection limit of 0.29 μmol (equal to 14.5 mM in 20 μL extracted liquid from solid sample). Although the detection limit was not the lowest among the reported glyphosate sensors, it is still applicable for testing glyphosate in some real samples. For example, FDA sets a residual limit of 20–200 mg kg^−1^ for soybean-related products (after solvent extraction, glyphosate residue in the liquid sample is at mM level). In addition, researchers should consider testing the glyphosate concentration in the pesticide products.

The spiked recoveries of glyphosate in the tap water and soybeans were measured to be 92–111% and 100–117%, respectively, giving a total spiked recovery in the range of 92 to 117%, suggesting such MIP-based fluorescent paper sensors had a high accuracy. Additionally, the cost of one piece of the fluorescent MIP-coated paper was around 0.6 RMB and were therefore much cheaper than the commercial glyphosate test strip (30 RMB per piece).

## 4. Conclusions

In this work, fluorescent MIPs targeting glyphosate were prepared using three kinds of amino-functionalized silanes as functional monomers based on the catalyst-free imprinting polymerization. Under the experimental conditions, the fluorescent MIP prepared by APTES showed a better selectivity than the MIPs with the use of AAPTMS and AAAPTMS. Thus, the imprinting polymerization mixture based on APTES was successfully used for the easy production of fluorescent MIP-coated paper sensor. Such fluorescent paper sensor could specifically recognize glyphosate rapidly (in 5 min) with a detection limit of 0.29 μmol, and it also had a much lower producing cost. However, although the fluorescent MIP-coated paper sensor was constructed successfully, there are still much work required before it the products can become efforts to devote commercially available, such as in paper size and fluorophore types, both of which can affect the sensitivity obviously; in pre-polymerization contents, which can affect the selectivity; in polymerization conditions that will determine the coating layer morphology and affect the detection speed and sensitivity, etc.

## Figures and Tables

**Table 1:** Content of the polymerization mixture for MIP particles.

Polymer Name	Template, 0.1 mmol	Functional Monomer, 0.6 mmol	Fluorescent Functional Monomer, 2 μmol
MIP1	Glyphosate	APTES	APTES-FITC
NIP1	/	APTES	APTES-FITC
MIP2	Glyphosate	AAPTMS	AAPTMS-FITC
NIP2	/	AAPTMS	AAPTMS-FITC
MIP3	Glyphosate	AAAPTMS	AAAPTMS-FITC
NIP3	/	AAAPTMS	AAAPTMS-FITC

Note: TEOS in the content is 3 mmol.

**Table 2:** Performances of MIPs for sensing glyphosate.

MIP	IF	Cross-Reactivity Factor	Analysis Time	LOD	Linear Range	Ref.
Guanidinium dyes-based fluorescent MIP particle	1.9	2.1, 2.1	2 min	4.8/0.6 µM	7.9–40.8 µM	[41]
MIP@Au electrochemical sensor	14.5	7.9, 43.5, 14.5	30 min	5.9 × 10^−6^ nM	1.8 × 10^−3^–296 nM	[44]
MIP@nanotube electrochemical sensor	8	4.1, 5.8, 6.1, 8	5 min	11.4 nM	14.8–2071 nM	[21]
MIP nanoparticle-coated electrochemical sensor	/	/	/	4.0 nM	0.025–500 mM	[45]
Fluorescent MIP mesoporous silica particles	2.9	1.4	2–3 min	1.45 µM	5–55 µM	[42]
Mn–ZnS QDs-based MIP-modified paper sensor	/	/	5 min	11.83 nM	29.6 nM–296 µM	[15]
Graphene QDs-based fluorescent MIP nanoparticle	/	/	/	0.1 nM	0–800 µM	[43]
MIP-based microfluidic electrochemical sensor	/	/	15 s	247/188 nM	0–50 µM	[46]
Inorganic framework MIP-based on Ni nanorod arrays	2.2	/	/	3.1 nM	0.01–1 µM	[47]
Polypyrrole MIP electrochemical sensor	9	/	18 min	1.6 µM	0.03–4.73 µM	[48]
MIP@ Au and Prussian Blue electrochemical sensor	3	2, 2.1, 4.3, 4	10 min	0.5 µM	2.4–7.1 µM	[31]
Polypyrrole MIP-based gravimetric and electrochemical sensors	/	/	30 min	1 pM	1 pM–1 nM	[49]
Polypyrrole MIP-based electrochemical surface plasmon resonance sensor	2.8	/	5 min	1.1/3.4 nM	0.05–0.5 mM	[50]
Fluorescent MIP silica particle	7.6	2.0, 8.2, 5.7	5 min	0.41 µM	0.5–20 μM	This work
Fluorescent MIP-coated paper sensor	2.0	2.0, 1.5, 2.8	5 min	0.29 µmol	0.5–10 μmol	This work

## Data Availability

No new data were created.

## Sample Availability

Samples of the compounds and materials are available from the authors.

## References

[1] Gomez-Caballero A., Diaz-Diaz G., Bengoetxea O., Quintela A., Unceta N., Goicolea M.A., Barrio R.J. (2016). Water compatible stir-bar devices imprinted with underivatised glyphosate for selective sample clean-up. J. Chromatogr. A.

[2] Santos J.S., Pontes M.S., Santiago E.F., Fiorucci A.R., Arruda G.J. (2020). An efficient and simple method using a graphite oxide electrochemical sensor for the determination of glyphosate in environmental samples. Sci. Total Environ..

[3] Do M.H., Florea A., Farre C., Bonhomme A., Bessueille F., Vocanson F., Tran-Thi N.-T., Jaffrezic-Renault N. (2015). Molecularly imprinted polymer-based electrochemical sensor for the sensitive detection of glyphosate herbicide. Int. J. Environ. Anal. Chem..

[4] Xu J., Smith S., Smith G., Wang W., Li Y. (2019). Glyphosate contamination in grains and foods: An overview. Food Control.

[5] Saunders L.E., Pezeshki R. (2015). Glyphosate in Runoff Waters and in the Root-Zone: A Review. Toxics.

[6] Rigobello-Masini M., Pereira E.A.O., Abate G., Masini J.C. (2019). Solid-Phase Extraction of Glyphosate in the Analyses of Environmental, Plant, and Food Samples. Chromatographia.

[7] Zouaoui F., Bourouina-Bacha S., Bourouina M., Alcacer A., Bausells J., Jaffrezic-Renault N., Zine N., Errachid A. (2020). Experimental Study and Mathematical Modeling of a Glyphosate Impedimetric Microsensor Based on Molecularly Imprinted Chitosan Film. Chemosensors.

[8] Wirth M.A., Schulz-Bull D.E., Kanwischer M. (2021). The challenge of detecting the herbicide glyphosate and its metabolite AMPA in seawater—Method development and application in the Baltic Sea. Chemosphere.

[9] Gotti R., Fiori J., Bosi S., Dinelli G. (2019). Field-amplified sample injection and sweeping micellar electrokinetic chromatography in analysis of glyphosate and aminomethylphosphonic acid in wheat. J. Chromatogr. A.

[10] Berry C. (2020). Glyphosate and cancer: The importance of the whole picture. Pest Manag. Sci..

[11] Zouaoui F., Bourouina-Bacha S., Bourouina M., Zine N., Errachid A., Jaffrezic-Renault N. (2022). Mathematical Modelling of Glyphosate Molecularly Imprinted Polymer-Based Microsensor with Multiple Phenomena. Molecules.

[12] (2021). National Standard of China. National Food Safety Standard-Maximum Residue Limits for Pesticides in Food.

[13] European Food Safety A. (2018). Review of the existing maximum residue levels for glyphosate according to Article 12 of Regulation (EC) No 396/2005. Eur. Food Saf. Auth. EF.

[14] Perez-Mayan L., Castro G., Ramil M., Cela R., Rodriguez I. (2022). Approaches to liquid chromatography tandem mass spectrometry assessment of glyphosate residues in wine. Anal. Bioanal. Chem..

[15] Sawetwong P., Chairam S., Jarujamrus P., Amatatongchai M. (2021). Enhanced selectivity and sensitivity for colorimetric determination of glyphosate using Mn–ZnS quantum dot embedded molecularly imprinted polymers combined with a 3D-microfluidic paper-based analytical device. Talanta.

[16] Ibarra Bouzada L.M.E., Hernández S.R., Kergaravat S.V. (2019). Glyphosate detection from commercial formulations: Comparison of screening analytic methods based on enzymatic inhibition. Int. J. Environ. Anal. Chem..

[17] Viirlaid E., Ilisson M., Kopanchuk S., Mäeorg U., Rinken A., Rinken T. (2019). Immunoassay for rapid on-site detection of glyphosate herbicide. Environ. Monit. Assess..

[18] Sanchís J., Kantiani L., Llorca M., Rubio F., Ginebreda A., Fraile J., Garrido T., Farré M. (2012). Determination of glyphosate in groundwater samples using an ultrasensitive immunoassay and confirmation by on-line solid-phase extraction followed by liquid chromatography coupled to tandem mass spectrometry. Anal. Bioanal. Chem..

[19] Vaghela C., Kulkarni M., Haram S., Aiyer R., Karve M. (2018). A novel inhibition based biosensor using urease nanoconjugate entrapped biocomposite membrane for potentiometric glyphosate detection. Int. J. Biol. Macromol..

[20] Ding X., Yang K.L. (2013). Development of an oligopeptide functionalized surface plasmon resonance biosensor for online detection of glyphosate. Anal. Chem..

[21] Ding S., Lyu Z., Li S., Ruan X., Fei M., Zhou Y., Niu X., Zhu W., Du D., Lin Y. (2021). Molecularly imprinted polypyrrole nanotubes based electrochemical sensor for glyphosate detection. Biosens. Bioelectron..

[22] Majdinasab M., Daneshi M., Louis Marty J. (2021). Recent developments in non-enzymatic (bio)sensors for detection of pesticide residues: Focusing on antibody, aptamer and molecularly imprinted polymer. Talanta.

[23] Li Y., Wang Y., Pei Y., Fan Z., Cao J., Wu J. (2018). Application of immunoassay techniques in glyphosate residue detection. Chin. J. Immunol..

[24] Melnikov P., Bobrov A., Marfin Y. (2022). On the Use of Polymer-Based Composites for the Creation of Optical Sensors: A Review. Polymers.

[25] Tanwar S., Mathur D. (2021). Graphene-based nanocomposites as sensing elements for the electrochemical detection of pesticides: A review. J. Solid State Electrochem..

[26] Li Q., Kamra T., Ye L. (2016). Nanoparticle-enhanced fluorescence emission for non-separation assays of carbohydrates using a boronic acid-alizarin complex. Chem. Commun..

[27] Li Q., Jiang L., Kamra T., Ye L. (2018). Synthesis of fluorescent molecularly imprinted nanoparticles for turn-on fluorescence assay using one-pot synthetic method and a preliminary microfluidic approach. Polymer.

[28] Pan J., Chen W., Ma Y., Pan G. (2018). Molecularly imprinted polymers as receptor mimics for selective cell recognition. Chem. Soc. Rev..

[29] Ye L., Mosbach K. (2008). Molecular Imprinting: Synthetic Materials As Substitutes for Biological Antibodies and Receptors. Chem. Mater..

[30] Xing R.R., Guo Z.C., Lu H.F., Zhang Q., Liu Z. (2021). Molecular imprinting and cladding produces antibody mimics with significantly improved affinity and specificity. Sci. Bull..

[31] Xu J., Zhang Y., Wu K., Zhang L., Ge S., Yu J. (2017). A molecularly imprinted polypyrrole for ultrasensitive voltammetric determination of glyphosate. Microchim. Acta.

[32] Wang H., Da L., Yang L., Chu S., Yang F., Yu S., Jiang C. (2020). Colorimetric fluorescent paper strip with smartphone platform for quantitative detection of cadmium ions in real samples. J. Hazard. Mater..

[33] Mampallil D., Eral H.B. (2018). A review on suppression and utilization of the coffee-ring effect. Adv. Colloid Interface Sci..

[34] Liu Y., Huang C.Z., Li Y.F. (2002). Fluorescence assay based on preconcentration by a self-ordered ring using berberine as a model analyte. Anal. Chem..

[35] Wang T., Li Q., Wang M., Xu J., Li J., Wang F. (2023). Synthesis of fluorescent artificial receptors with high specificity for simultaneous detection of non-steroidal anti-inflammatory drugs. Food Chem..

[36] Wang F., Wang D., Wang T., Jin Y., Ling B., Li Q., Li J. (2021). A simple approach to prepare fluorescent molecularly imprinted nanoparticles. RSC Adv..

[37] Jin Y., Wang T., Li Q., Wang F., Li J. (2022). A microfluidic approach for rapid and continuous synthesis of glycoprotein-imprinted nanospheres. Talanta.

[38] Xu R., Dai S., Dou M., Yang J., Wang X., Liu X., Wei C., Li Q., Li J. (2023). Simultaneous, Label-Free and High-throughput SERS Detection of Multiple Pesticides on Ag@Three-Dimensional Silica Photonic Microsphere Array. J. Agric. Food Chem..

[39] Wang F., Ling B., Li Q., Abouhany R. (2020). Dual roles of 3-aminopropyltriethoxysilane in preparing molecularly imprinted silica particles for specific recognition of target molecules. RSC Adv..

[40] Ye L., Cormack P.A.G., Mosbach K. (2001). Molecular imprinting on microgel spheres. Anal. Chim. Acta.

[41] Kimani M., Pérez-Padilla V., Valderrey V., Gawlitza K., Rurack K. (2022). Red-Emitting Polymerizable Guanidinium Dyes as Fluorescent Probes in Molecularly Imprinted Polymers for Glyphosate Detection. Chemosensors.

[42] Kimani M., Kislenko E., Gawlitza K., Rurack K. (2022). Fluorescent molecularly imprinted polymer particles for glyphosate detection using phase transfer agents. Sci. Rep..

[43] Kim Y., Lee J., Shin I.S. (2019). Advanced method for fabrication of molecularly imprinted mesoporous organosilica with highly sensitive and selective recognition of glyphosate. Sci. Rep..

[44] Zouaoui F., Bourouina-Bacha S., Bourouina M., Abroa-Nemeir I., Ben Halima H., Gallardo-Gonzalez J., El Alami El Hassani N., Alcacer A., Bausells J., Jaffrezic-Renault N. (2020). Electrochemical impedance spectroscopy determination of glyphosate using a molecularly imprinted chitosan. Sens. Actuators B Chem..

[45] Thimoonnee S., Somnet K., Ngaosri P., Chairam S., Karuwan C., Kamsong W., Tuantranont A., Amatatongchai M. (2022). Fast, sensitive and selective simultaneous determination of paraquat and glyphosate herbicides in water samples using a compact electrochemical sensor. Anal. Methods.

[46] Uka B., Kieninger J., Urban G.A., Weltin A. (2021). Electrochemical Microsensor for Microfluidic Glyphosate Monitoring in Water Using MIP-Based Concentrators. ACS Sens..

[47] Zhao Y., Yan Y., Liu C., Zhang D., Wang D., Ispas A., Bund A., Du B., Zhang Z., Schaaf P. (2022). Plasma-Assisted Fabrication of Molecularly Imprinted NiAl-LDH Layer on Ni Nanorod Arrays for Glyphosate Detection. ACS Appl. Mater. Interfaces.

[48] Zhang C., She Y., Li T., Zhao F., Jin M., Guo Y., Zheng L., Wang S., Jin F., Shao H. (2017). A highly selective electrochemical sensor based on molecularly imprinted polypyrrole-modified gold electrode for the determination of glyphosate in cucumber and tap water. Anal. Bioanal. Chem..

[49] Mazouz Z., Rahali S., Fourati N., Zerrouki C., Aloui N., Seydou M., Yaakoubi N., Chehimi M.M., Othmane A., Kalfat R. (2017). Highly Selective Polypyrrole MIP-Based Gravimetric and Electrochemical Sensors for Picomolar Detection of Glyphosate. Sensors.

[50] Balciunas D., Plausinaitis D., Ratautaite V., Ramanaviciene A., Ramanavicius A. (2022). Towards electrochemical surface plasmon resonance sensor based on the molecularly imprinted polypyrrole for glyphosate sensing. Talanta.

